# Comparison between vascular Doppler ultrasound and contrast exams in chronic peripheral arterial disease

**DOI:** 10.1590/1677-5449.202301042

**Published:** 2024-07-08

**Authors:** Alex Aparecido Cantador, Ana Terezinha Guillaumon

**Affiliations:** 1 Universidade Estadual de Campinas – Unicamp, Hospital de Clínicas – HC, Campinas, SP, Brasil.; 2 Universidade Estadual de Campinas – Unicamp, Faculdade de Ciências Médicas – FCM, Campinas, SP, Brasil.

**Keywords:** Doppler ultrasound, peripheral arterial disease, angiotomography, endovascular, iodinated contrast

## Abstract

**Background:**

Vascular Doppler ultrasound (DUS) has evolved over recent years because of improvements in the technology involved in the acquisition and processing of sound and image data. The method is an excellent option for use in diagnosis of peripheral arterial disease considering its availability, low cost, and absence of harmful effects. The breakdown of logistics supply chains caused by the COVID-19 pandemic caused worldwide shortages of iodinated contrast, highlighting the need to validate alternative diagnostic methods.

**Objective:**

To use DUS for decision-making when choosing between by-pass and endovascular surgery for femoropopliteal arterial disease and compare the results to those of iodinated contrast exams.

**Methods:**

We compared DUS with examinations using contrast for identification of stenoses/occlusions and indication of surgical treatment (by-pass vs. endovascular). In the first phase of the study the results were merely compared, DUS vs. angiotomography. Then, in the second phase, the vascular ultrasound results were used for screening between by-pass and endovascular treatment, comparing DUS with angiotomography in cases scheduled for by-pass and with arteriography in endovascular patients.

**Results:**

In phase 1, the sensitivity of DUS compared to CT angiography was 100% for the SFA territory. When considering solely the choice of bypass vs. endovascular treatment, the results showed 100% agreement for phase 1 and 94% for phase 2.

**Conclusion:**

Notwithstanding the sample size, the study fulfilled its objective of demonstrating the reliability of DUS for indicating the treatment choice between by-pass and endovascular surgery.

## INTRODUCTION

The imaging exams routinely used for morphological analysis and definition of interventional treatment for peripheral arterial occlusive disease are arteriography or angiotomography, both of which involve exposure to radiation and iodinated contrast.^1-3^ Against this background, vascular echography has been used successfully in patients with peripheral arterial disease (PAD).^4-8^ For the femoropopliteal axis, and particularly for assessment of the superficial femoral artery (SFA), it achieves high levels of agreement with the gold standard diagnostic method (arteriography), although discrepancies are larger in the infragenicular territory.^9-12^

Noninvasive diagnostic methods have improved over recent years, as technology has advanced. Vascular ultrasonography can be used to assess the dynamics of arterial flow (wave patterns, velocities, points of turbulence, etc.) combining information from Doppler with morphological assessment, expanding the range of information available for decision-making.^11^ Advantages of using vascular Doppler ultrasound (DUS) as a screening method include its cost-effectiveness and noninvasiveness, with no need for exposure to radiation or iodinated contrast. Disadvantages include its examiner-dependent characteristics and difficulties with using it in obese patients.^10-13^

According to the 2017 European Society of Cardiology Guidelines, DUS is indicated as first-line examination for confirmation of PAD (recommendation class I, evidence level C).^11^ Additionally, many centers use DUS for arterial assessment and for planning treatment in peripheral occlusive disease, but there are few references in the literature reporting validation of the method. One study of relevance to the issue was published in 2021 by García-Rivera et al.,^14^ demonstrating that DUS had 80% sensitivity and 95.45% specificity in femoropopliteal lesions when compared with intraoperative angiography. These results suggest that DUS could merit consideration as the only preoperative imaging exam for surgical planning in patients scheduled for lower limb revascularization procedures. Martinelli et al.^15^ studied 94 patients with PAD and compared DUS with angiotomography for the aortoiliac, femoropopliteal, and infragenicular axes, showing good diagnostic agreement for the femoropopliteal territory (Cohen’s K: 0.93 - 0.96).

The objective of this study is to compare DUS with examinations employing contrast (angiotomography or arteriography) in cases of PAD involving the femoropopliteal segment, analyzing sensitivity, specificity, and predictive values. The study also aims to present a rational management approach to choosing a contrast imaging method, taking into consideration the most likely treatment suggested by the DUS results.

## METHODS

This study was submitted to and approved by the Research Ethics Committee, decision number 3.850.999 on February 20, 2020, under Ethics Appraisal Submission Certificate 28632619.9.0000.5404.

This is a comparative study of diagnostic tests (vascular ultrasound vs. examinations using contrast) in patients with PAD involving the femoropopliteal segment, defined according to presence of trophic ulcers or pain at rest, with femoral pulse present, popliteal absent, and distal pulses absent. A total of 30 consecutive patients were recruited prospectively at a tertiary teaching hospital affiliated to the Unified Health System (SUS - Sistema Único de Saúde, SUS). The study design was defined prior to data collection (prospectively) and employs double-blinding (ultrasound examiners were blind to the results of the examinations using contrast and the results had no impact on clinical management of patients). The sample was limited to 30 individuals for reasons of convenience with respect to the study procedures.

Patients who refused to participate were excluded, at no detriment to their treatment, as were patients with chronic renal failure with serum creatinine exceeding 2.0 mg/dL.

No adverse events were recorded during the investigation, bearing in mind that echography does not involve risk to subjects’ health. On the contrary, it is an additional tool for diagnosis that offers the benefit of avoiding unnecessary angiotomography examinations. This avoids unnecessary doses of radiation and contrast and reduces health care costs.

All participants signed a free and informed consent form and were assured that their voluntary and confidential participation would not interfere with treatment, considering the methods described below.

Our routine conduct for cases of chronic arterial obstruction with trophic ulcers or pain at rest, with femoral pulse present, and popliteal and distal pulses absent, is to conduct angiotomography and then decide on conventional surgical treatment (by-pass) or an endovascular intervention. The most important parameter on which this decision is based is the anatomic presentation of stenosis and obstructions in the femoropopliteal segment. Cases in which the origin of the SFA is compromised, or in which the popliteal artery is isolated or there is occlusion of the popliteal artery at the joint line have lower probability of successful endovascular treatment and are routinely treated with by-pass.

The objective of this study is to use DUS as an alternative to angiotomography for initial screening and, depending on the results, refer the patient for diagnostic angiotomography or for arteriography with the possibility of conducting endovascular treatment during the procedure.

**Figure 1:**
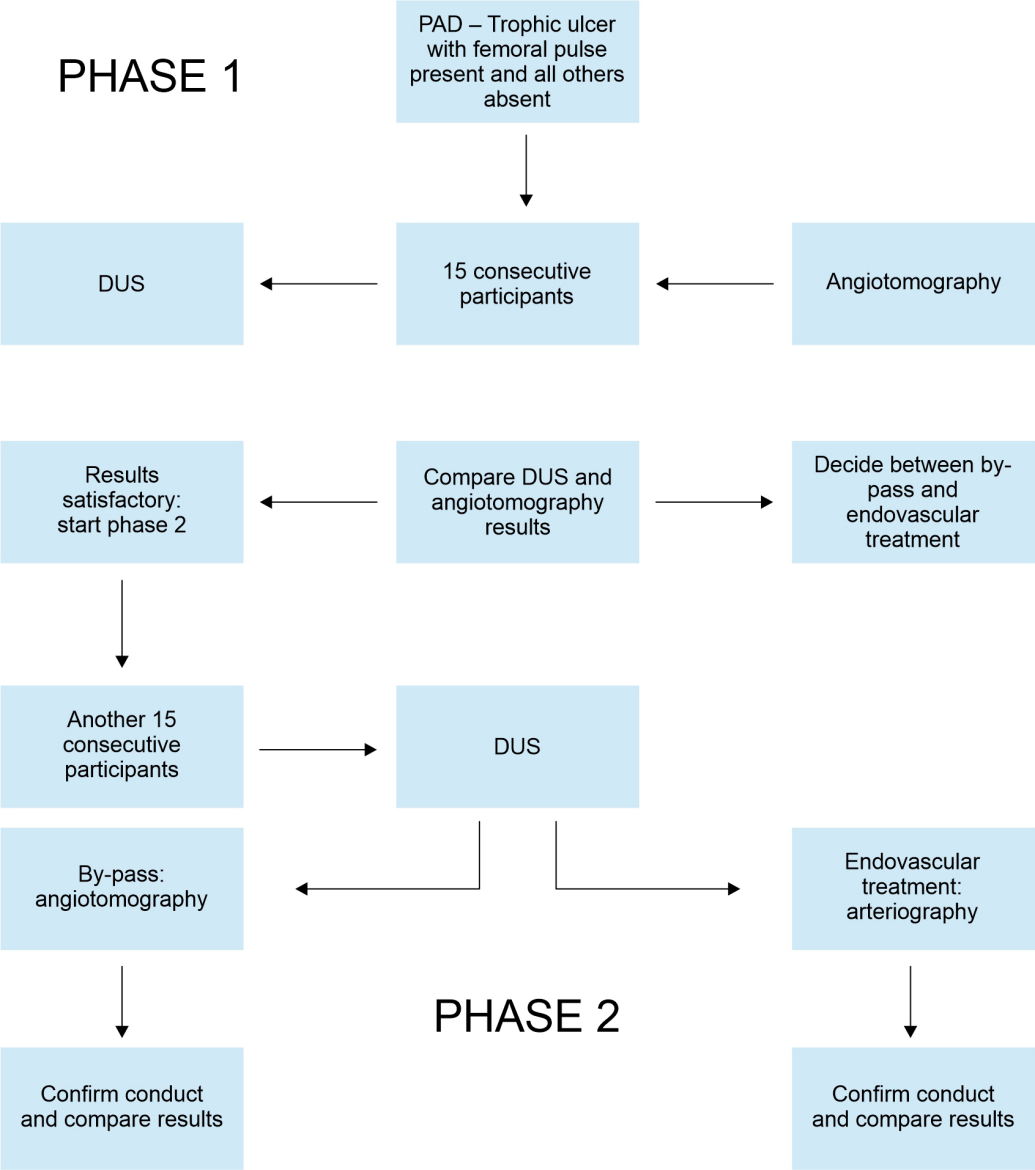
Flow diagram demonstrating patient selection and the division between the two study phases. PAD = Peripheral arterial disease; DUS = Vascular Doppler ultrasound.

During Phase 1, ultrasound was conducted, but was not yet used to define treatment (i.e., angiotomography was also performed in all cases, according to the service’s routine practice) and the DUS and angiotomography findings were compared. All DUS examinations were performed by assistant physicians who were certified to conduct DUS by the Brazilian College of Radiology and the Brazilian Society of Angiology and Vascular Surgery. The treatment proposal was defined on the basis of the angiotomography findings after other assistant physicians from the vascular surgery team had interpreted the angiotomography images for this purpose. The results were then compared blind to determine sensitivity, specificity, and positive and negative predictive values. The Phase 1 sample was defined as 15 patients. This study did not involve any risk to patients, since the treatment routine was not changed because of the study and the additional DUS examination was not used to guide the choice of treatment. The study was divided into two phases so that DUS could be tested in Phase 1 without impacting on patient management. Once it had been confirmed that Phase 1 results were satisfactory, it was possible to move on to the second phase, in which DUS was used to guide referral of the patient for angiotomography or arteriography with the intention to treat, as illustrated in Figure 1.

In Phase 2, the DUS results guided the decision on the next step, i.e., they were used to indicate angiotomography or arteriography with a proposal for endovascular treatment during the procedure. If the DUS findings were compatible with endovascular treatment, the patient would be referred directly for angiography with the intention to treat, with no need for angiotomography. If the arteriography results did not agree with the DUS findings and were indicative of a need for by-pass surgery rather than endovascular treatment, the patient would be referred for the appropriate treatment. In contrast, patients whose DUS results were suggestive of a probable need for by-pass surgery would be referred for angiotomography and conventional surgery, i.e., the service’s routine management would be maintained. This phase recruited a second sample of 15 patients and the DUS findings were once more compared with the results of arteriography and angiotomography producing data on sensitivity, specificity, and predictive values.

Our sample was restricted to 15 consecutive patients in each phase, for reasons of convenience and to make execution of the study feasible. However, the ideal sample size, calculated using SAS (Statistical Analysis System, SAS Institute Inc, United States), applying a Cohen’s kappa coefficient of 0.5 (the minimum reasonable) and considering an estimated 70% agreement between DUS and angiotomography, would be 79 individuals in each phase.

## RESULTS

Contingency tables (2×2) were populated with frequencies (absolute and percentages) considering the DUS results compared to the examinations using contrast. The McNemar test was used to determine agreement between the tests and simple kappa coefficients, sensitivity, specificity, accuracy, positive predictive value (PPV), negative predictive value (NPV), positive likelihood ratio, and negative likelihood ratio were calculated.

A 5% significance level was adopted for this study.

In Phase 1, the sensitivity of DUS for diagnosis of occlusion and stenosis in the SFA territory was 100%, achieving 100% agreement when compared with angiotomography. In Phase 2, the sensitivity of DUS when compared with the examinations using contrast was 75% (Confidence Interval [CI] 35.5-95.5%), with specificity 100%, PPV 100%, NPV 77.78% (CI40.1-96.0%), negative likelihood ratio 0.25 (CI0.07-0.83), and kappa coefficient 0.73.

For the popliteal artery, the Phase 1 results were sensitivity 85.71% (IC42-99%), specificity 87.5% (IC46-99%), PPV 85.71% (IC42-99%), NPV 87.5% (IC46-99%), negative likelihood ratio 0.16% (IC0.02-1.02%) and kappa coefficient 0.73. The Phase 2 result for the popliteal artery was 100% agreement between DUS and the examinations using contrast.

These results are presented in Table 1, showing the SFA and popliteal artery territories.

**Table 1:** Results for the SFA (superficial femoral artery) and popliteal artery in Phase 1 and Phase 2.

Territory	Sensitivity	Specificity	PPV	NPV
SFA (Phase 1)	100%	100%	100%	100%
SFA (Phase 2)	75%	100%	100%	77.78%
Popliteal (Phase 1)	85.71%	87.5%	85.71%	87.5%
Popliteal (Phase 2)	100%	100%	100%	100%

PPV = positive predictive value; NPV = negative predictive value.

These results relate to presence of hemodynamically significant stenosis or occlusions in each artery. However, if we consider only the indications for treatment with by-pass or endovascular techniques, agreement in Phase 1 between DUS and examinations using contrast was 100% and agreement in Phase 2 was 94%.

No complications were recorded in relation to this study, whether due to angiotomography (contrast allergy or impaired renal function) or angiography (pseudoaneurysms, bleeding, embolization, or others).

## DISCUSSION

The consequences of the COVID-19 pandemic, with breakdown of logistics chains, stoppages at contrast production centers in China, and logjams at ports globally, caused worldwide shortages of iodinated contrast. Governmental authorities issued warnings to health services, recommending use of alternative diagnostic methods to tomography with contrast, including a recommendation to only use contrast for urgent and emergency procedures. This episode illustrates another consequence of the pandemic health problems for the population, demonstrating the need for alternative diagnostic methods to those needing contrast.

Although the sensitivity of DUS for diagnosis of stenosis and occlusions in the SFA territory was 75% in Phase 2, when the indications for treatment (by-pass vs. endovascular) were analyzed, agreement was 100% in Phase 1 and 94% in Phase 2. These results confirm that DUS is a very effective method for initial diagnostic assessment to select candidates for endovascular treatment, with greater than 90% accuracy, as demonstrated in our results and other similar results available in the literature. Before by-pass surgery or endovascular treatment, an examination using contrast should be performed to confirm the correct decision and the intervention method chosen, reducing the possibility of error based on the diagnosis by DUS. Patients with DUS results compatible with endovascular treatment would undergo arteriography with an intention to treat, thereby avoiding unnecessary use of angiotomography and reducing exposure to contrast and radiation. In contrast, patients with DUS results compatible with a need for by-pass surgery will undergo angiotomography for confirmation (following the standard routine at the service).

This study’s sample size was small and further studies should be undertaken to obtain additional evidence and propose protocols for making a rational decision on the choice between angiotomography and arteriography based on DUS findings and for planning treatment.

Despite its insufficient sample size, this study suggests that vascular ultrasound is highly accurate for choosing between endovascular treatment and by-pass surgery for cases of PAD involving the femoropopliteal axis. The sample sizes of future studies investigating the subject should be larger. Regardless, the advantages of vascular ultrasound in terms of cost-benefit and the absence of harmful effects justify its use, supporting decision-making and avoiding unnecessary angiotomography examinations in cases eligible for endovascular treatment, reducing costs and contrast usage.

## CONCLUSIONS

We conclude that DUS achieves good sensitivity and specificity in comparison with examinations using contrast for assessment of PAD involving the femoropopliteal segment. It also achieves high accuracy for indicating the most likely treatment, guiding the choice of examination with contrast for confirmation, notwithstanding the small sample size of this study.
