# Supplementary material for: Comparison between vascular Doppler ultrasound and contrast exams in chronic peripheral arterial disease
Source: J Vasc Bras. 2024 Jul 8;23:e20230104. doi: 10.1590/1677-5449.202301042 (PMC11296684; doi:10.1590/1677-5449.202301042)
Supplement: Translation

# Translation: Comparação entre ecografia vascular com Doppler e exames contrastados na doença arterial crônica periférica

**DOI:** 10.1590/1677-5449.202301041

**Authors:** Alex Aparecido Cantador, Ana Terezinha Guillaumon

**Affiliations:** 1 Universidade Estadual de Campinas – Unicamp, Hospital de Clínicas – HC, Campinas, SP, Brasil.; 2 Universidade Estadual de Campinas – Unicamp, Faculdade de Ciências Médicas – FCM, Campinas, SP, Brasil.

**Keywords:** ecografia vascular com Doppler, doença arterial periférica, angiotomografia, endovascular, contraste iodado

## Abstract

**Contexto:**

A ecografia vascular com Doppler (EVD) evoluiu nos últimos anos devido ao aprimoramento da tecnologia de aquisição e processamento da imagem. A disponibilidade do exame, o baixo custo e a ausência de efeitos deletérios de radiação e contraste tornam este método uma excelente opção no diagnóstico da doença arterial periférica. As quebras nas cadeias de suprimentos devido à pandemia de covid-19 levaram a uma escassez global de contraste iodado, reforçando a importância de validar abordagens alternativas.

**Objetivos:**

Utilizar a EVD na decisão entre cirurgia aberta ou endovascular para doença arterial femoropoplítea e comparar os resultados com exames de contraste iodado.

**Métodos:**

Comparamos EVD com exames contrastados (angiotomografia e arteriografia) em relação à localização de estenoses/oclusões e indicação do tratamento cirúrgico (*by-pass* vs. endovascular). Em uma primeira fase, os resultados foram apenas comparados entre EVD e angiotomografia. Numa segunda fase, os resultados da EVD foram usados na triagem entre *by-pass* vs. endovascular), sendo comparados com angiotomografia nos casos de cirurgia aberta e comparados com a arteriografia nos casos de tratamento endovascular.

**Resultados:**

A sensibilidade da EVD em comparação com a angiotomografia na fase 1 foi de 100% para o território da artéria femoral superficial. Ao considerar apenas a indicação de *by-pass* *versus* endovascular, os resultados mostraram 100% de concordância para a Fase 1 e 94% para a Fase 2.

**Conclusões:**

Com a ressalva do tamanho amostral, o estudo cumpriu seu objetivo de demonstrar a confiabilidade da EVD na indicação do tratamento cirúrgico entre aberto ou endovascular.

## INTRODUÇÃO

Os exames de imagem rotineiramente utilizados para análise morfológica e definição do tratamento intervencionista na doença arterial periférica são a arteriografia ou a angiotomografia, ambas envolvendo exposição à radiação e contraste iodado^1-3^ . Dentro desse contexto, a ecografia vascular tem sido aplicada com sucesso em pacientes com doença arterial periférica (DAP)^4-8^ . No eixo fêmoro-poplíteo, particularmente para avaliação da artéria femoral superficial (AFS), apresenta alta concordância com o método diagnóstico padrão-ouro (arteriografia), sendo o território infragenicular o que apresenta maior discrepância^9-12^ .

Com os avanços tecnológicos dos últimos anos, os métodos diagnósticos não invasivos evoluíram muito. A ultrassonografia vascular permite a avaliação dinâmica do fluxo arterial (padrões de ondas, velocidades, pontos de turbulência, etc) combinando as informações do Doppler com a avaliação morfológica e aumentando o leque de informações disponíveis para a tomada de decisões^11^ . Entre as vantagens do uso da ecografia vascular com Doppler (EVD) como método de triagem estão a custo-efetividade do método e sua abordagem não invasiva, sem o uso de radiação e contraste iodado. Entre as desvantagens estão a característica examinador-dependente e as dificuldades da utilização em doentes obesos^10-13^ .

De acordo com a Diretriz da Sociedade Europeia de Cardiologia de 2017, a EVD é indicada como exame de primeira linha na confirmação da DAP (classe I, nível de evidência C)^11^ . Além disso, vários centros utilizam a EVD para avaliação arterial e planejamento do tratamento na doença oclusiva periférica, mas há poucas referências para a validação do método na literatura. Um estudo relevante na área, publicado em 2021 por García-Rivera et al.^14^ , demonstrou uma sensibilidade de 80% e especificidade de 95,45% em lesões fêmoro-poplíteas para a EVD em comparação com a angiografia intraoperatória. Os resultados sugerem que a EVD pode ser considerada como um exame de imagem pré-operatório único durante o planejamento cirúrgico em pacientes submetidos a um procedimento de revascularização de membros inferiores. Martinelli et al.^15^ estudaram 94 pacientes com DAP e compararam a EVD com a angiotomografia nos eixos aorto-ilíaco, fêmoro-poplíteo e abaixo do joelho, mostrando boa concordância diagnóstica no eixo fêmoro-poplíteo (K de Cohen 0,93 - 0,96).

O presente trabalho tem como objetivo comparar a EVD com os exames contrastados (angiotomografia ou arteriografia) nos casos de DAP do segmento fêmoro-poplíteo, avaliando sensibilidade, especificidade e valores preditivos. Além disso, busca apresentar uma alternativa de abordagem racional na escolha do exame contrastado, considerando o tratamento provável sugerido pelo resultado da EVD.

## MÉTODOS

Esta pesquisa foi submetida ao Comitê de Ética em Pesquisa, com parecer favorável sob o número 3.850.999 de 20/02/2020, com o número de Certificado de Apresentação de Apreciação Ética 28632619.9.0000.5404.

O estudo realizado é uma comparação entre testes diagnósticos (ecografia vascular vs. exames contrastados) em pacientes com DAP acometendo o segmento fêmoro-poplíteo, definidos pela presença de lesão trófica ou dor em repouso, com pulso femoral presente, poplíteo ausente e distais ausentes. Foram estudados prospectivamente 30 doentes consecutivos em hospital terciário de ambiente universitário do Sistema Único de Saúde (SUS). O delineamento do estudo foi realizado anteriormente à coleta dos dados (prospectivo) e duplo-cego (examinadores de ecografia sem conhecimento dos resultados dos exames contrastados e sem interferência na condução clínica dos doentes). A amostra foi limitada a 30 indivíduos por razão de conveniência na execução da pesquisa.

Foram excluídos os doentes que se recusaram a participar da pesquisa, sem prejuízo de seu tratamento, e os portadores de insuficiência renal crônica com creatinina sérica superior a 2,0 mg/dL.

Não foi registrado nenhum evento adverso no decorrer da investigação, tendo em vista que a ecografia não oferece riscos à saúde dos participantes. Trata-se de uma ferramenta complementar no diagnóstico, proporcionando o benefício de poupar exames de angiotomografia desnecessários. Dessa forma, evitam-se doses desnecessárias de radiação e contraste, reduzindo custos para o serviço de saúde.

Todos os participantes assinaram o Termo de Consentimento Livre e Esclarecido, sendo-lhes garantido o sigilo de sua participação voluntária sem comprometer ou interferir no tratamento, considerando os métodos descritos a seguir.

Nossa rotina nos casos de obstrução arterial crônica com lesão trófica ou dor em repouso, com pulso femoral presente e pulsos poplíteo e distais ausentes, é a realização de angiotomografia, seguida da decisão pelo tratamento cirúrgico convencional (*by-pass*) ou endovascular. O principal parâmetro para essa decisão é a apresentação anatômica de estenoses e obstruções no segmento fêmoro-poplíteo, sendo que, casos com comprometimento da origem da AFS, ou que apresentam artéria poplítea isolada, ou ainda oclusão da artéria poplítea na interlinha articular, têm menor probabilidade de sucesso no tratamento endovascular e são rotineiramente tratados de forma convencional (*by-pass*).

O objetivo deste estudo é realizar EVD em alternativa a angiotomografia para triagem inicial e, dependendo do resultado, encaminhar o doente para angiotomografia ou arteriografia diagnóstica com possibilidade de tratamento endovascular ao mesmo tempo.

Na Fase 1 foi realizada a ecografia, ainda que não utilizada como exame para definição do tratamento. Ou seja, foi realizada angiotomografia concomitante em todos os casos, seguindo a rotina normal do serviço, e realizada comparação entre EVD e angiotomografia. Todos os exames de EVD foram realizados por médicos assistentes certificados na área de EVD pelo Colégio Brasileiro de Radiologia e pela Sociedade Brasileira de Angiologia e Cirurgia Vascular. A proposta de tratamento foi realizada baseada na angiotomografia, cujas imagens foram interpretadas por outros médicos assistentes da equipe de cirurgia vascular. Os resultados foram comparados cegamente, considerando sensibilidade, especificidade e valores preditivos positivos e negativos. A amostra da Fase 1 foi definida em 15 doentes. Nesta fase do estudo não houve risco para o doente, tendo em vista que a rotina de tratamento não foi alterada pela pesquisa, pois o exame agregado da EVD não orientou a definição do tratamento. O trabalho foi dividido em duas fases, de modo a testar a EVD na Fase 1 sem interferir na conduta dos pacientes. Com os resultados da Fase 1 satisfatórios, foi possível passar para a segunda fase, em que a EVD definiu o direcionamento do doente para angiotomografia ou arteriografia com proposta terapêutica, como mostra a Figura 1.

**Figura 1:** Fluxograma demonstrando a seleção dos doentes e a divisão entre as duas fases do estudo. DAP = Doença arterial periférica; Fe+ = Pulso femoral presente; EVD = Ecografia vascular com Doppler.

Na Fase 2, os resultados da EVD nortearam a definição da etapa seguinte, ou seja, indicaram a realização de angiotomografia ou arteriografia com proposta de tratamento endovascular concomitante. Caso os achados da EVD fossem sugestivos de tratamento endovascular, o doente seria encaminhado diretamente à angiografia com proposta terapêutica, sem necessidade de realização de angiotomografia. Caso a arteriografia apresentasse resultado discrepante da EVD, com indicação de *by-pass* ao invés de tratamento endovascular, o doente seria encaminhado para o tratamento adequado. Por outro lado, os pacientes avaliados por EVD com provável indicação de *by-pass* foram encaminhados para angiotomografia e cirurgia convencional, ou seja, mantendo a rotina habitual do serviço. Nesta fase, utilizando uma nova amostra de 15 doentes, comparamos a EVD com os resultados da arteriografia e da angiotomografia, novamente obtendo dados de sensibilidade, especificidade e valores preditivos.

Nossa amostra foi limitada a 15 doentes consecutivos em cada fase, por questão de conveniência e plausibilidade na execução do estudo. No entanto, a amostra ideal calculada, de acordo com o software SAS (Statistical Analysis System, SAS Institute Inc, EUA), aplicado no coeficiente kappa de Cohen de 0,5 (mínimo razoável), deveria ser de 79 indivíduos para cada fase, considerando a concordância estimada de 70% entre a EVD e a angiotomografia.

## RESULTADOS

Foram produzidas tabelas de contingência (2×2) com valores de frequências (absolutas e percentuais) considerando a EVD em relação ao exame contrastado. Para avaliar a concordância entre os testes utilizou-se o teste de McNemar e foram obtidas as medidas de coeficiente kappa simples, sensibilidade, especificidade, acurácia, valor preditivo positivo (VPP), valor preditivo negativo (VPN), razão de verossimilhança positiva e razão de verossimilhança negativa.

O nível de significância adotado para o estudo foi de 5%.

Na Fase 1, a sensibilidade da EVD para diagnosticar oclusão e estenose no território da AFS foi de 100%, mostrando 100% de concordância em comparação com a angiotomografia. Na Fase 2, a sensibilidade da EVD em comparação com o exame contrastado mostrou 75% (IC35,5-95,5%), especificidade 100%, VPP 100%, VPN 77,78% (IC40,1-96,0%). Razão de verossimilhança negativa de 0,25 (IC0,07-0,83) e coeficiente kappa 0,73.

Para a artéria poplítea, os resultados na Fase 1 foram: sensibilidade 85,71% (IC42-99%), especificidade 87,5 (IC46-99%), VPP 85,71% (IC42-99%), VPN 87,5% (IC46-99%), razão de verossimilhança negativa de 0,16% (IC0,02-1,02%) e coeficiente kappa 0,73. Os resultados da Fase 2 para a poplítea foram de 100% de concordância entre a EVD e o exame contrastado.

Os resultados são apresentados na Tabela 1, mostrando os territórios da AFS e da artéria poplítea.

**Tabela 1:** Resultados para AFS (artéria femoral superficial) e artéria poplítea para Fase 1 e Fase 2.

Território	Sensibilidade	Especificidade	VPP	VPN
AFS (Fase 1)	100%	100%	100%	100%
AFS (Fase 2)	75%	100%	100%	77,78%
Poplítea (Fase 1)	85,71%	87,5%	85,71%	87,5%
Poplítea (Fase 2)	100%	100%	100%	100%

VPP = valor preditivo positivo; VPN = valor preditivo negativo.

Os resultados apresentados contemplam a presença de estenoses hemodinamicamente significativas ou oclusões em cada artéria especificamente. Por outro lado, se considerarmos apenas a indicação de tratamento *by-pass* ou endovascular, na Fase 1 houve 100% de concordância entre a EVD e o exame contrastado, e 94% de concordância na Fase 2.

Nenhuma complicação foi relacionada a este estudo, nem com a angiotomografia (alergia ao contraste ou comprometimento da função renal), nem com a angiografia (pseudoaneurismas, sangramento, embolização, entre outros).

## DISCUSSÃO

As consequências da pandemia de covid-19, com a quebra de cadeias logísticas, bloqueios em centros produtores de contraste na China, e congestionamentos em portos em todo o mundo levaram à escassez global de contraste iodado. As autoridades governamentais emitiram alertas aos serviços de saúde recomendando o uso de métodos diagnósticos alternativos à tomografia com contraste, incluindo a recomendação de usar contraste apenas em urgências e emergências. Este episódio retrata mais uma das consequências dos problemas de saúde da pandemia para a população, demonstrando a necessidade de métodos diagnósticos alternativos ao contraste.

Embora os resultados da sensibilidade da EVD para o diagnóstico de estenoses e oclusões no território da AFS na Fase 2 tenham sido de 75%, quando considerada a indicação de tratamento (*by-pass* vs. endovascular), a concordância foi de 100% na Fase 1 e de 94% na Fase 2. Esses resultados confirmam que a EVD é um método muito eficiente na abordagem diagnóstica inicial na seleção de candidatos ao tratamento endovascular, tendo mais de 90% de acurácia, conforme demonstrado por nossos resultados e outros semelhantes disponíveis na literatura. Antes da cirurgia de *by-pass* ou do tratamento endovascular, deve ser realizado um exame contrastado para a correta decisão e confirmação do método intervencionista, reduzindo a possibilidade de erro baseado no diagnóstico por EVD. Os doentes com resultados de EVD elegíveis para tratamento endovascular seriam submetidos a arteriografia com proposta terapêutica, evitando uso desnecessário da angiotomografia e reduzindo a exposição ao contraste e à radiação. De outra forma, os doentes com resultados de EVD elegíveis para *by-pass* seriam submetidos à angiotomografia de confirmação (seguindo a rotina do serviço). O tamanho da amostra deste estudo é limitado e novos estudos devem ser feitos para obter mais evidências e propor protocolos para escolha racional entre angiotomografia ou arteriografia com base nos achados da EVD e no planejamento do tratamento.

Apesar do tamanho amostral insuficiente, este trabalho sugere alta acurácia da ecografia vascular na escolha entre o tratamento endovascular e *by-pass* para os casos de DAP do eixo fêmoro-poplíteo. O tamanho amostral pode ser aumentado em futuros estudos neste tema. Além disso, as vantagens da ecografia vascular em relação ao custo-benefício e ausência de efeitos deletérios justificam sua utilização, auxiliando na tomada de decisão e evitando exames de angiotomografia desnecessários para os casos de tratamento endovascular, reduzindo custos e uso de contraste.

## CONCLUSÃO

Concluímos que a EVD apresenta bons parâmetros de sensibilidade e especificidade na comparação com os exames contrastados para avaliação da DAP do segmento fêmoro-poplíteo. Além disso, apresenta alta acurácia na indicação do provável tratamento, norteando a escolha do exame contrastado de confirmação, com a ressalva do tamanho amostral limitado do estudo.
